# Differences between centers in functional outcome of patients with ADHD after 1 year from the time of diagnosis

**DOI:** 10.1038/s41598-023-45714-y

**Published:** 2023-10-31

**Authors:** Massimo Cartabia, Stefano Finazzi, Maurizio Bonati, The Lombardy ADHD Group, Maurizio Bonati, Massimo Cartabia, Nicoletta Raschitelli, Michele Zanetti, Stefano Conte, Valeria Renzetti, Patrizia Stoppa, Valentina Mauri, Massimo Molteni, Antonio Salandi, Sara Trabattoni, Paola Effredi, Elisa Fazzi, Elena Filippini, Elisabetta Pedercini, Alessandra Tiberti, Patrizia Conti, Elena Della Libera, Nadia Fteita, Maria Teresa Giarelli, Giacomo Piccini, Luciano Viola, Simona Frassica, Federico Ravaglione, Stefania Villa, Daniela Alacqua, Ottaviano Martinelli, Davide Villani, Emanuela Binaghi, Matteo Caletti, Andrea Deriu, Gabriella Vasile, Giada Ariaudo, Paola Morosini, Barbara Salvatore, Maddalena Breviglieri, Giuseppe Capovilla, Chiara Galantini, Gaia Oldani, Vera Valenti, Chiara Battaini, Emiddio Fornaro, Alessandra Valentino, Aglaia Vignoli, Jessica Babboni, Claudio Bissoli, Antonella Costantino, Isabella Cropanese, Anna Didoni, Laura Reale, Maria Paola Canevini, Ilaria Costantino, Valentina Tessarollo, Mauro Walder, Elisa Baroffio, Renato Borgatti, Matteo Chiappedi, Connie Capici, Maria Luisa Carpanelli, Maria Grazia Palmieri, Gianpaolo Ruffoni, Sara Mometti, Francesco Rinaldi, Federica Soardi, Giorgio Rossi, Carla Sgrò, Cristiano Termine

**Affiliations:** 1 Laboratory of Pharmacoepidemiology, Department of Public Health, Istituto di Ricerche Farmacologiche Mario Negri IRCCS, Milan, Italy; 2 Laboratory of Clinical Data Science, Department of Public Health,, Istituto di Ricerche Farmacologiche Mario Negri IRCCS, Ranica, BG Italy; 3 Laboratory for Mother and Child Health, Department of Public Health, Istituto di Ricerche Farmacologiche Mario Negri IRCCS, Via Mario Negri, 2, 20156 Milano, Italy; 4 Azienda Ospedaliera “Papa Giovanni XXII”, Bergamo, Italy; 5 Eugenio Medea, Associazione La Nostra Famiglia IRCCS, Bosisio Parini, LC Italy; 6 Spedali Civili, Brescia, Italy; 7 Ospedale “S. Anna”, Como, Italy; 8 Azienda Istituti Ospitalieri, Cremona, Italy; 9 Azienda Ospedaliera “G. Salvini”, Garbagnate Milanese, MI Italy; 10 Ospedale “A. Manzoni”, Lecco, Italy; 11 Ospedale Civile, Legnano, MI Italy; 12 Azienda Ospedaliera, Lodi, Italy; 13 Azienda Ospedaliera “C. Poma”, Mantova, Italy; 14 Azienda Ospedaliera “Fatebenefratelli”, Milan, Italy; 15 Azienda Ospedaliera Niguarda Cà Granda, Milan, Italy; 16 Fondazione IRCCS Cà Granda Ospedale Maggiore Policlinico, Milan, Italy; 17 Azienda Ospedaliera Santi Carlo e Paolo, Milan, Italy; 18 IRCCS Mondino, Pavia, Italy; 19 Azienda Ospedaliera della Valtellina e della Valchiavenna, Sondrio, Italy; 20 ASL Vallecamonica-Sebino, Esine, BS Italy; 21 Ospedale “Del Ponte”, Varese, Italy

**Keywords:** Health care, Medical research

## Abstract

Although the pharmacological therapy of ADHD has been widely studied, little has been done to compare the different therapeutic approaches (e.g., drug therapy vs. psychological treatments) and even less has been done to compare the outcome of the therapy between centers. This multicenter observational study aims to assess between-center variation in functional outcome of ADHD patients one year after the diagnosis, according to the treatment received. We used the Regional ADHD Registry data on 1429 patients enrolled in 16 ADHD centers in the 2011–2022 period. To evaluate the effectiveness of the therapy we used a generalized linear mixed model with the center as the random effect, including patient condition at diagnosis and center characteristics, weighting by the inverse of the propensity score of the treatment received by the patient. Between-center variation was expressed as the relative difference in odds-ratios between the observed and the expected number of patients whose condition improved, using the Clinical Global Impressions—Improvement Scale (CGI-I), and the relative 95% CI. Patients who received combined treatment were significantly more likely to improve compared to other treatment groups (65.5% vs 54.4% for methylphenidate alone, 53.4% for psychological treatment alone, or 40.5% for no therapy). Adjusted for patients and center characteristics, the log-odds ratio ranged from 0.85 (0.29–1.55 95% CI) to − 0.64 (− 1.17–− 0.18 95% CI). The mean expected probability of improvement after one year of therapy for an average patient with ADHD for each center was 47.7% in a center at the 25th percentile and 61.2% in a center at the 75th percentile of the outcome distribution after adjustments. The wide between-center variation in patient functional improvement one year after the diagnosis of ADHD could be largely explained by center-specific therapeutic approaches or attitudes. More careful and stringent work is needed to reduce differences in responses between centers, as could formal and periodic audit programs within and between centers.

## Introduction

Attention-deficit/hyperactivity disorder (ADHD) is a common behavioral condition and common chronic illness in children. Much research has occurred during last decade increasing the understanding of ADHD and contributing to the setting up of appropriate clinical resources for the evaluation, diagnosis, and treatment of the disorder already in its early manifestation^1,2^. This neurodevelopmental disorder affects 5.9% of children and persists into adulthood for two-thirds of cases, with risk of impairments in academic achievement and work^3^. The core symptoms are inattention, restlessness, and impulsivity and are more frequent in boys than girls (ratio 3:1). The prevalence of the disorder ranges from 1.1 to 3.1% of the pediatric population, considering only subjects with a diagnosis confirmed by clinical evaluation^4^. Despite the acquired knowledge, several barriers hamper effective and timely diagnosis and treatment of patients with ADHD^1,5^. These barriers include limited access to care because of inadequate number, organization, structure, and staffing of child mental health services, lack of professional updating on managing the disorder, and the fragmentation of care. It has also been described that Factors beyond health care access and unequal symptom levels seem responsible for the geographical variation in ADHD diagnosis^6^.

A project aiming to ensure appropriate ADHD management for children and adolescents once the disorder is suspected was activated over a decade ago in the Lombardy Region, the first Italian Region for population density and productivity^7,8^. The intense and fruitful work done so far has been widely reported^4,7–15^, documenting some of the variables that contribute the most to the efficacy of both clinical^14^ and organizational care^15^. From a clinical and policy standpoint, the main aim of the project was to enable all ADHD Regional centers acting as specialized ADHD hubs (tier three) to guarantee high-quality care for children and adolescents evaluated and treated for ADHD. Systematic, collaborative efforts were made by all the ADHD centers belonging to the Lombardy ADHD Group over the years, creating and sharing a rigorous diagnostic and therapeutic protocol, and including even the creation of the Regional ADHD Registry. Worldwide, healthcare systems and practices need to be organized with a strong focus on measuring and improving outcomes of care. The quality and efficacy of care, also for ADHD patients, need to be guaranteed through the evaluation of outcomes. It is well-known in medicine that both patient and center characteristics can affect patient outcome. Little is known about the between-center variation in outcomes in patients treated for ADHD. Variation in outcomes between ADHD centers can be caused by differences in patient population (e.g., age, ADHD severity), but also by structural differences (e.g., staffing), or differences in processes (e.g., time to diagnosis). Center-specific structural and process factors are largely modifiable, contrary to patient characteristics. Insight into modifiable factors that could explain between-center variation in outcomes may inform ADHD work processes and thereby improve patient recovery. In this study, we aim to assess the between-center variation in outcome, as related to clinical evaluation of ADHD patients, after one year of therapy from the time of diagnosis, measured by the CGI-Improvement (CGI-I) scale, adjusting for patient and center characteristics.

## Methods

A retrospective study based on medical records was conducted. Data were identified from the Regional ADHD Registry. The study wa approved by the Institutional Review Board of the Istituto di Ricerche Farmacologiche Mario Negri IRCCS, Milan, Italy. Written informed consent from the parents of participants was obtained before data collection. We used the previously described methodology and reported data concerning the local health setting, the characteristics of the ADHD Registry activated in Lombardy in June 2011, the systematic work carried out by the 18 ADHD centers, and the diagnostic assessment and the treatment conducted by all involved clinicians^4,7–15^, according to the national and international guidelines^16,17^. In over a decade of study, every month the Coordination Center sent a newsletter free of charge to all participants and interested parties with the update of the scientific publications that appeared in the international literature in the previous month on the subject of ADHD. Upon request, the Coordination Center also provided the PDF of the paper requested to the interested clinician. The necessary steps were clinical anamnestic and psychiatric interview, neurological examination, evaluation of cognitive level by Wechsler Scales^18–20^, the schedule for affective disorders and schizophrenia for school-age children (K-SADS)^21^, and the Developmental and Well-Being Assessment (DAWBA)^22^. Behavioral and emotional problems were highlighted with the most used and validated rating scales for parents and teachers, Conners’ Parent Rating Scale revised (CPRS-R)^23^, Conners’ Teacher Rating Scale revised (CTRS-R)^24^, and the Child Behavior Checklist (CBCL)^25^, while symptom severity was quantified with the use of the Clinical Global Impressions—Severity (CGIS)^26^. The Clinical Global Impressions—Improvement Scale (CGI-I) scores were analyzed after 12–18 months of follow-up. Data on the annual activity of the 18 ADHD centers (annual hours of work per patient on the project and number of dedicated operators) were collected with an annual ad-hoc survey as part of the project, and median values for each center and for each of the years 2018, 2019 and 2020, were recorded. Then we computed the median values of the center medians. Eventually, for each variable we created two categories: less or equal to this median, or greater than the median.

The type of therapy received by the patients was classified into 4 categories:

- None: no medications taken for ADHD therapy, no cycle of psychological therapy undertaken between the diagnosis and the follow-up visit;
- Only pharmacological: at least one prescription and use of methylphenidate between the diagnosis and the follow-up visit;
- Only psychological: at least one performed psychological treatment (child training, cognitive, speech therapy, parent training, psychodynamic, psychomotricity, teacher training, family therapy) between the diagnosis and the follow-up visit;
- Combined: methylphenidate and psychological treatment between the diagnosis and the follow-up visit.

Every 6 months the working group met for a collegial evaluation and any updates to the diagnostic-therapeutic path based on findings of the work and the results of published clinical research.

Data were extracted from the database and analyses were updated on 1 February 2022. Data referred to patients added between 2011 and 2021. We considered only the patients of the centers with at least 10 evaluable patients present in the register, and excluded the few patients receiving atomoxetine or other drugs different from methylphenidate, since methylphenidate is the first-line drug with a specific indication for ADHD treatment in < 18 year-old patients in Italy.

### Data analyses

All data were entered in a SAS/STAT database (SAS Version 9.4, SAS Institute, Inc., Cary, NC, USA). Descriptive statistics were computed for the entire study population and for subgroups. Kruskall-Wallis and Chi-square tests were used to compare the characteristics of patients among the centers, a p-value of less than 0.05 was considered as significant. We provided a descriptive table of patients’ characteristics and treatments by improvement. Differences were evaluated with Chi-square test or Wilcoxon test. To evaluate treatment efficacy, we also calculated standardized residuals (Std. Res)^27^. A standardized residual is the difference between the observed and expected values for a single treatment group: the larger the residual, the greater the contribution of the group to the magnitude of the resulting Chi-square obtained value. To evaluate the effectiveness of the therapy we used a generalized linear mixed model with center as random effect, including patient conditions at diagnosis and center characteristics, weighting by the inverse of the propensity score of the treatment performed by the patient. The propensity score was built through a multinomial logistic regression model using the treatment as outcome and the characteristics of patients as independent variables^28^. A Chi-square test was performed to evaluate the homogeneity of the weights among the four groups of therapy. To evaluate the differences between centers, we calculated the odds-ratios between the observed and the expected number of patients with improvements and the relative 95% CI. We used two different methods to evaluate the expected number of patients with improvements. First, we computed the raw expected number of patients with improvements in each center as the number of patients treated in the center multiplied by the average fraction of patients with improvements computed on the whole sample. Then, three generalized linear mixed models with center as random effect were developed, to evaluate the determinants of the difference between centers at different levels: in the first model (M1) we adjusted by patient characteristics, in the second model (M2) we added the characteristics of the center, and in the third model (M3) we also added the type of therapy given. For each model, we computed the adjusted expected number of patients with improvements in each center by summing the probabilities of improvement estimated for all patients treated by the center. Finally, we calculated the log-odds ratios between the observed and the adjusted expected number of patients with improvements and the relative 95% CI. Then we calculated the mean expected probability of improvement after one year of therapy for each center, assuming that all the observed patients were followed by that center. First we estimated a generalized linear mixed models with center as the random effect, then we used the coefficients of that model to estimate the probability of improvement on a new dataset by replacing the center characteristic of each patient with the adjusted estimates. Then we calculated the mean probability on all the observations.

The results are presented as the number, frequency (%), and mean or median; *p* < 0.05 was considered to be significant.

### Ethics approval

Formal ethical review board approval was not required for the present analysis of the data. The present research was approved by the Institutional Review Board of the IRCCS Istituto di Ricerche Farmacologiche “Mario Negri” in Milan, Italy. Parental consent was obtained for all the participants before data collection.

### Consent to participate

All the participants in the study have given written informed consent at the time of recruitment. Data were anonymised prior to use for research purposes. The analysis in the current study is approved by the members of the Lombardy ADHD Group and were performed (as well as all procedures) in accordance with relevant guidelines/regulations as recommended by the Institutional Review Board.

## Results

### Descriptive analyses

A total of 1429 children and adolescents, from 16 centers, with ADHD diagnosed for the first time and clinically evaluated after a one-year period were included in this study. These children and adolescents, 86% male, had a median age of 8 years at the diagnosis (range 5–17 years) for a median of 59 (range of 24–252) youths per center (Table 1). The range of median time from the time of request to the diagnosis was 50–370 days 162 overall). Of the 1429 children and adolescents in the study, 996 (70%) had at least one comorbid psychiatric disorder, and 80 (5.6%) had a chronic medical disease. 911 of 1429 patients (63.8%) had ADHD of the combined type, 393 (27.5%) of the inattentive type, and 125 (8.7%) of the hyperactive/impulsive type. At baseline parents consistently rated their children higher than teachers on the Cognitive Problems/Inattention subscale (B) of the CPRS, with a number of participants with scores within the pathological range according to the parents’ ratings that was significantly larger than that calculated from CTRS teachers’ answers (CPRS-B, n = 883, 73.2%; CTRS-B, n = 706, 58.5%; *p* < 0.0001). Comparison of the rates using the Hyperactivity (C) subscale (CPRS-C, n = 784, 65%; CTRS-C, n = 837, 69,3%; *p* = 0.0061) and the Emotional Lability (J) subscale (CPRS-J, n = 472, 39,1%; CTRS-J, n = 566, 46,9%; *p* < 0.0001) yielded different results, with a higher rate of participants’ scores in the pathological range when rated by teachers than by parents. At follow-up parents and teachers rated children more closely maintain a slight difference for CPRS-B subscale (CPRS-B, n = 213, 73.2%; CTRS-B, n = 566, 57.3%; *p* < 0.0312). Comorbid psychiatric disorders were more frequent in patients with ADHD of combined type (OR 1.40 IC 1.11–1.77) and in those with a CGI-S score equal to or greater than 5 (OR 2.22, IC 1.70–2.90). Half of patients (52.6%) received only psychological treatment, and 16% of patients combined treatment. Methylphenidate alone was taken by 180 patients (12.6%), whereas 18.8% of patients did not receive any therapy. A wide variability between centers was found for all considered variables.

**Table 1:** Characteristics of the ADHD centers and patients.

Characteristics	Total	Range between centers	*p*
***Centers* (N = 16)**			
Clinical staff professionals (median)	5	2–11	
Hours/year/patient of work (median)	10.0	4.0–70.4	
Median time from the request to diagnosis (days)	162	50–370	< 0.0001*
***Patients* N (%)**			
Total	1.429	24–252	
Median age at diagnosis (years)	8	8—11	< 0.0001*
Male	1.232 (86.2)	19–210 (77.4–97.9)	0.0002*
Only child	368 (25.8)	4–70 (11.4–34.9)	0.0813
Born abroad	73 (5.1)	0–18 (0–11.5)	0.0072*
Adopted	58 (4.1)	0–13 (0–9.5)	0.0642
School failures	44 (3.1)	0–11 (0–8.6)	0.1564
Employed parents	911 (63.8)	14–154 (40.0–83.3)	< 0.0001*
Family history of ADHD	269 (18.8)	3–54 (7.3–34.0)	< 0.0001*
Dystocic delivery	338 (23.7)	4–47 (11.4–38.5)	0.0640
Preterm/low weight	149 (10.4)	1–20 (2.9–20,4)	0.0907
Exclusive breastfeeding ≥ 3 months	732 (51.2)	15–116 (9.6–76.9)	< 0.0001*
Motor delay	73 (5.1)	1–9 (1.3–17.1)	0.0196*
Language delay	315 (22.0)	2–56 (7.5–31.9)	0.0049*
** ADHD type**			
Combined	911 (63.8)	10–180 (28.6–83.9)	< 0.0001*
Inattentive	393 (27.5)	7–68 (14.3–48.2)	< 0.0001*
Hyperactive/impulsive	125 (8.7)	1–26 (1.5–31.4)	< 0.0001*
CGI-S score 5–7 at diagnosis	448 (31.4)	5–81 (5.7–70.8)	< 0.0001*
**Comorbidities**			
Psychiatric comorbidities	996 (69.7)	17–173 (48.9–95.0)	< 0.0001*
Chronic diseases	80 (5.6)	0–14 (0–29.2)	< 0.0001*
***Therapy* N (%)**			
Psychological alone	751 (52.6)	3–135 (12.5–84.9)	< 0.0001*
Combined	229 (16.0)	1–50 (2.1–42.9)	< 0.0001*
Methylphenidate alone	180 (12.6)	0–43-(0–50.0)	< 0.0001*
None	269 (18.8)	2–75 (3.2–51.2)	< 0.0001*

• Test χ^2^ used for categorical variables and Kruskall-Wallis for continuous variables.

*Statistically significant.

### Between-center variation in outcome

Clinical outcome evaluation of ADHD patients after one year of therapy, as measured by the CGI-I scale, was evaluated. Overall, 758 (53.0%) patients showed improvement.

Findings of the Chi-square test for each center or patient characteristics (counting the number of improved patients, CGI-I ≤ 3) showed no statistical difference between improvement and considered variables with the exception of treatment variable (*p* < 0.0001) and yearly hours of work per patient above the median (*p* = 0.02) (Supplementary Table 1). Patients who received combined treatment were significantly more likely to improve compared to other treatment groups (65.5% vs. 54.4% for methylphenidate alone, 53.4% for psychological treatment alone, and 40.5% for no therapy). Residual analysis confirmed that the greatest contribution treatment to the Chi-square test’s significance was by the combined group, whose subjects were significantly more likely to improve compared to other treatment groups (Supplementary Fig. 1).

The effectiveness of the treatment strategies was assessed using a logistic regression model weighted by the inverse of the propensity of receiving the treatment (Supplementary Table 2). The use of propensity score results in more numerically treatment balanced groups (*p* = 0.50, Supplementary Table 3). Combined therapy performed better than the other therapies (OR: 4.61, 95%CI 3.87–5.49), and was followed by methylphenidate alone (OR: 2.70, 95%CI 2.27–3.21), and psychological therapy alone (OR: 2.05, 95%CI 1.75–2.41) (Table 2).

**Table 2:** Effectiveness of the therapy.

Characteristics	OR (IC 95%)	*p*
***Centers***		
Clinical staff professionals over the median: No versus Yes	1.00 (0.90–1.12)	0.9628
Hours/year/patient of work over the median: No versus Yes	1.13 (0.63–2.03)	0.6824
Median time from the request to diagnosis (months)	1.00 (1.00–1.00)	0.0002*
***Patients***		
Age at diagnosis	0.98 (0.96–1.01)	0.1501
Sex: Male versus Female	0.88 (0.74–1.05)	0.1539
Only child: Yes versus No	1.09 (0.95–1.24)	0.2155
Born abroad: Yes versus No	0.53 (0.39–0.71)	< 0.0001*
Adopted child: Yes versus No	0.66 (0.47–0.93)	0.0173*
School failures: Yes versus No	0.66 (0.48–0.90)	0.0083*
Employed parents: Yes versus No	0.79 (0.70–0.90)	0.0002*
Family history of ADHD: Yes versus No	1.09 (0.94–1.28)	0.2500
Dystocic delivery: Yes versus No	0.97 (0.84–1.12)	0.6829
Preterm/low weight: Yes versus No	0.81 (0.68–0.98)	0.0261*
Exclusive breastfeeding (≥ 3 months): Yes versus No	1.13 (0.99–1.28)	0.0658
Motor delay: Yes versus No	2.45 (1.84–3.28)	< 0.0001*
Language delay Yes versus No	0.54 (0.47–0.62)	< 0.0001*
ADHD type H/I versus C	1.05 (0.93–1.20)	0.4158
CGI-S score at diagnosis 5–7 versus 3–4	0.95 (0.82–1.09)0.95 (0.83–1.10)	0.5116
**Comorbidities**		
Psychiatric comorbidities: Yes versus No	1.04 (0.87–1.24)	0.6619
Chronic diseases: Yes versus No	0.90 (0.69–1.17)	0.4356
***Therapy ***versus*** None***		
Combined	4.61 (3.87–5.49)	< 0.0001*
Methylphenidate alone	2.70 (2.27–3.21)	< 0.0001*
Psychological alone	2.05 (1.75–2.41)	< 0.0001*

Generalized linear mixed model with center as the random effect, adjusted for patient conditions at diagnosis.

and center characteristics, weighting by the inverse of the propensity of receiving the treatment.

**Figure 1:**
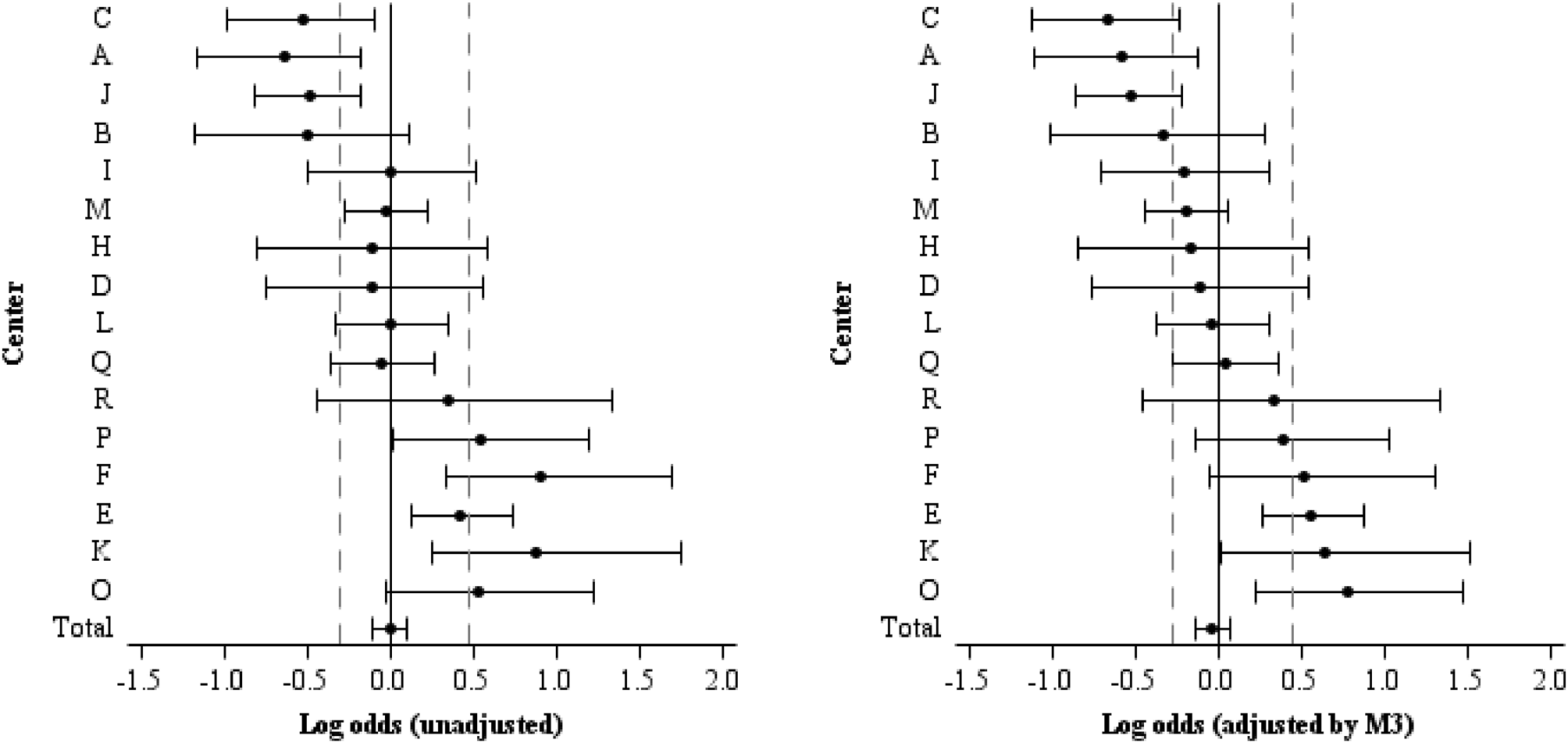
Log-odds ratios between observed and expected improvements. Unadjusted probability of improvement (left) and adjusted probability estimated by Model 3 (right). The dashed lines represent the 25th and 75th percentile of the improvement distribution.

When assessing whether patient improvement differed between centers of the three developed models, M3 was found to be the most explanatory (Supplementary Table 4). The log-odds ratios between observed and expected number of patients with improvements by center (Supplementary Table 5), and after the addition of patient characteristics at admission, center characteristics, and type of treatment received (M3), the relative difference in odds decreased (Supplementary Table 6). With these adjustments, centers P and E still performed better than expected and centers F and K performed as expected, while center O performed better than expected. Centers A, C and J still performed worse than expected (Fig. 1).

**Figure 2:**
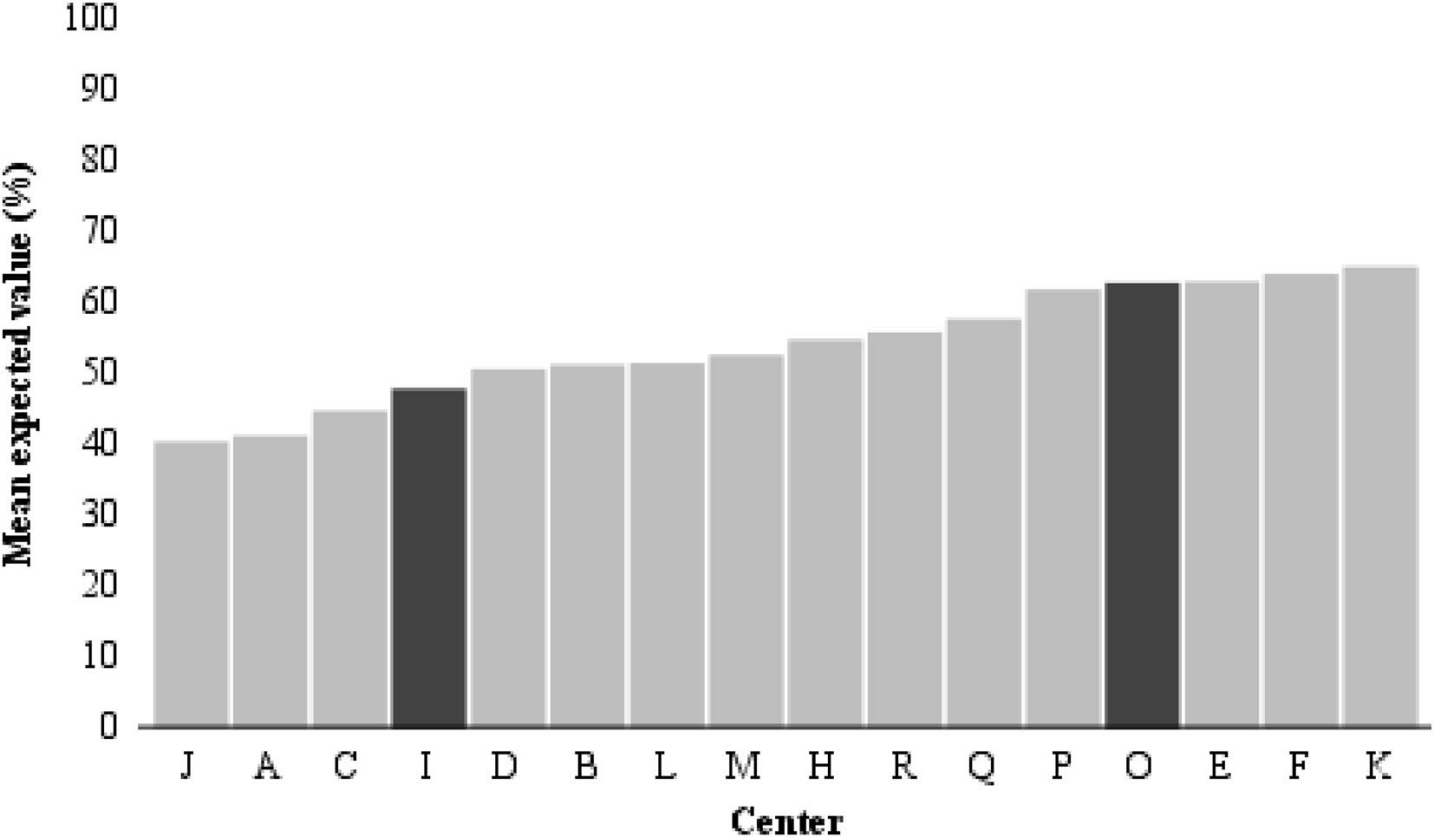
Estimated frequency of improvement for each center. The darkest bars represent the centers at the 25th and 75th percentile of the outcome distribution, corresponding to an interquartile range of the mean expected probabilities to achieve improvement of 48–63%, adjusted for patient and center characteristics.

The mean expected probability of improvement after one year of therapy for an average patient with ADHD for each center was 47.7% in a center at the 25^th^ percentile and 61.2% in a center at the 75^th^ percentile of the outcome distribution after adjustments (M3) (Fig. 2).

## Discussion

Improvement in ADHD patients is significantly better in subjects receiving combined therapy. The fraction of patients with improvements is lower in patients receiving only pharmacological or psychological treatment and significantly lower in those receiving no therapy. Findings, providing advantages for positive functioning outcomes measured by CGI scales (CGIs), are in agreement with those from the MTA study^29,30^. When pharmacological treatment is indicated, ADHD guidelines unanimously suggest the use of stimulants in children, in particular methylphenidate^31–33^. Likewise for psychosocial interventions, even though there is less agreement because the different types of interventions used are less well established^33^. Regarding the treatment, it is also necessary to consider the compliance between what is prescribed and what is taken or done. 18.8% of patients did not receive any therapy require both interventions in the provision of prescribed treatment by the public Centers and and further clarifications with some families.

Improvement results were adjusted by patient characteristics at diagnosis of ADHD and by center resources, and weighed by the inverse of the propensity of receiving the treatment in order to control biases caused by a possible imbalance between centers in the number of patients treated with a certain therapy or by correlations between patient severity and the choice of the treatment. Despite adjusting for patient and center characteristics and treatment, wide differences between centers in the outcomes. Moreover, this difference remained when the center performance (improved outcomes) was estimated considering the entire study population. This supports the validity of the results, and underlines the clinical and ethical implications for the public ADHD health services.

A plausible explanation of findings obtained may be that it is not only the patient or center characteristics that determine the differences between the centers, but that other variables, such as each teams’ experience and the updating of the approach and management of the disorder, that can improve outcomes, as reported for other clinical areas^34–36^. Differences in outcomes between centers that are due to different treatment policies or quality of care are undesirable, and must also be corrected by increasing adherence to guidelines. All this should be the subject of future studies aimed at identifying which variables of the real world of care influence the appropriateness.

Evaluating long-term clinical outcomes of ADHD as long-term effects of treatments, as efficacy and safety, has been challenging because of the difficulties in overcoming bias in studies and differences in practice. In the meantime, training interventions^37^ and clinical audits between and within ADHD centers could be performed to verify whether the services are performing well, and where improvements could be made to provide all patients with the best and most appropriate care available according to quality and equity principles^38–40^.

### Strengths and limitations

Variables related to patients and centers that were collected may not be sufficient to fully describe the patients’ conditions or the centers’ characteristics, but were among the reference standards for performing audits in clinical practice^38–40^. From the methodological point of view, the present study was an observational study conducted in the clinical practice context, not a randomized controlled trial (RCT) aimed at measuring the efficacy of different treatments. We used CGI scales to measure treatment outcome, knowing that they have their strengths and weaknesses^41^, because they are currently used in clinical practice. One year of follow up may be insufficient considering the numerous factors that can influence the outcome, but the severity of the disorder and the expectations of patients and families require a prompt and effective response. Chronicity, improvement, and recovery will, however, be monitored over time by the regional network as shown by the work of the MTA. Finally, the uniqueness of the present study makes it difficult to generalize the results to other regions and contests, however, their magnitude should be taken into account when redesigning ADHD systems.

## Conclusion

Although a holistic approach, using appropriate medication and psychological treatments, is the prevailing, evidence based indication for ADHD, large differences remained between reference ADHD centers. More careful and stringent work is needed to reduce differences in responses between centers, as could formal, periodic audit programs within and between centers. We sincerely hope that addressing and documenting differences between centers in outcomes will help to improve ADHD care.

### Supplementary Information

Supplementary Information.

## Data Availability

The datasets analyzed during the current study are available from the corresponding author upon reasonable request.
