# Author Correction: Differences between centers in functional outcome of patients with ADHD after 1 year from the time of diagnosis

**DOI:** 10.1038/s41598-023-48487-6

**Published:** 2023-12-15

**Authors:** Massimo Cartabia, Stefano Finazzi, Maurizio Bonati, The Lombardy ADHD Group, Maurizio Bonati, Massimo Cartabia, Nicoletta Raschitelli, Michele Zanetti, Stefano Conte, Valeria Renzetti, Patrizia Stoppa, Valentina Mauri, Massimo Molteni, Antonio Salandi, Sara Trabattoni, Paola Effredi, Elisa Fazzi, Elena Filippini, Elisabetta Pedercini, Alessandra Tiberti, Patrizia Conti, Elena Della Libera, Nadia Fteita, Maria Teresa Giarelli, Giacomo Piccini, Luciano Viola, Simona Frassica, Federico Ravaglione, Stefania Villa, Daniela Alacqua, Ottaviano Martinelli, Davide Villani, Emanuela Binaghi, Matteo Caletti, Andrea Deriu, Gabriella Vasile, Giada Ariaudo, Paola Morosini, Barbara Salvatore, Maddalena Breviglieri, Giuseppe Capovilla, Chiara Galantini, Gaia Oldani, Vera Valenti, Chiara Battaini, Emiddio Fornaro, Alessandra Valentino, Aglaia Vignoli, Jessica Babboni, Claudio Bissoli, Antonella Costantino, Isabella Cropanese, Anna Didoni, Laura Reale, Maria Paola Canevini, Ilaria Costantino, Valentina Tessarollo, Mauro Walder, Elisa Baroffio, Renato Borgatti, Matteo Chiappedi, Connie Capici, Maria Luisa Carpanelli, Maria Grazia Palmieri, Gianpaolo Ruffoni, Sara Mometti, Francesco Rinaldi, Federica Soardi, Giorgio Rossi, Carla Sgrò, Cristiano Termine

**Affiliations:** 1 Laboratory of Pharmacoepidemiology, Department of Public Health, Istituto di Ricerche Farmacologiche Mario Negri IRCCS, Milan, Italy; 2 Laboratory of Clinical Data Science, Department of Public Health,, Istituto di Ricerche Farmacologiche Mario Negri IRCCS, Ranica, BG Italy; 3 Laboratory for Mother and Child Health, Department of Public Health, Istituto di Ricerche Farmacologiche Mario Negri IRCCS, Via Mario Negri, 2, 20156 Milano, Italy; 4 Azienda Ospedaliera “Papa Giovanni XXII”, Bergamo, Italy; 5 Eugenio Medea, Associazione La Nostra Famiglia IRCCS, Bosisio Parini, LC Italy; 6 Spedali Civili, Brescia, Italy; 7 Ospedale “S. Anna”, Como, Italy; 8 Azienda Istituti Ospitalieri, Cremona, Italy; 9 Azienda Ospedaliera “G. Salvini”, Garbagnate Milanese, MI Italy; 10 Ospedale “A. Manzoni”, Lecco, Italy; 11 Ospedale Civile, Legnano, MI Italy; 12 Azienda Ospedaliera, Lodi, Italy; 13 Azienda Ospedaliera “C. Poma”, Mantova, Italy; 14 Azienda Ospedaliera “Fatebenefratelli”, Milan, Italy; 15 Azienda Ospedaliera Niguarda Cà Granda, Milan, Italy; 16 Fondazione IRCCS Cà Granda Ospedale Maggiore Policlinico, Milan, Italy; 17 Azienda Ospedaliera Santi Carlo e Paolo, Milan, Italy; 18 IRCCS Mondino, Pavia, Italy; 19 Azienda Ospedaliera della Valtellina e della Valchiavenna, Sondrio, Italy; 20 ASL Vallecamonica-Sebino, Esine, BS Italy; 21 Ospedale “Del Ponte”, Varese, Italy

Correction to: *Scientific Reports* 10.1038/s41598-023-45714-y, published online 31 October 2023

The original version of the Article contained an error in the Consortia author list of The Lombardy ADHD Group, where author name Gaia Oldani was incorrectly given as Gala Garden.

The original Article has been corrected.

