# Supplementary material for: Differences between centers in functional outcome of patients with ADHD after 1 year from the time of diagnosis
Source: Sci Rep. 2023 Oct 31;13:18738. doi: 10.1038/s41598-023-45714-y (PMC10618531; doi:10.1038/s41598-023-45714-y)

**Supplementary Table 1.** Characteristics of the ADHD centers and patients by clinical improvement (CGI-I ≤ 3)

| **Characteristics** | **Improved** | **Not**  **improved** | **Total** | **%**  **improved** | **p•** |  |
| --- | --- | --- | --- | --- | --- | --- |
| ***Centers*** |  |  |  |  |  |  |
| In centers with clinical staff professionals  over the median | 321 (42.3) | 292 (43.5) | 613 (42.9) | 52.4 | 0.6559 |  |
| In centers with hours/year/patient of work  over the median | 328 (43.3) | 250 (37.3) | 578 (40.4) | 56.7 | 0.0208 | * |
| Median time from the request to diagnosis (days) (IQR) | 155  (84 - 279) | 170  (94 - 289) | 162  (88 - 285) |  | 0.1116 |  |
| ***Patients* N (%)** |  |  |  |  |  |  |
| Total | 758 | 671 | 1.429 | 53.0 |  |  |
| Median age at diagnosis (years) (IQR) | 8 (7 - 10) | 8 (7-10) | 8 (7-10) |  | 0.9833 |  |
| Male | 644 (85.0) | 588 (87.6) | 1232 (86.2) | 52.3 | 0.1440 |  |
| Only child | 198 (26.1) | 170 (25.3) | 368 (25.8) | 53.8 | 0.7345 |  |
| Born abroad | 40 (5.3) | 33 (4.9) | 73 (5.1) | 54.8 | 0.7584 |  |
| Adopted | 33 (4.4) | 25 (3.7) | 58 (4.1) | 56.9 | 0.5484 |  |
| School failures | 26 (3.4) | 18 (2.7) | 44 (3.1) | 59.1 | 0.4143 |  |
| Employed parents | 495 (65.3) | 416 (62.0) | 911 (63.8) | 54.3 | 0.1944 |  |
| Family history of ADHD | 136 (17.9) | 133 (19.8) | 269 (18.8) | 50.6 | 0.3644 |  |
| Dystocic delivery | 186 (24.5) | 152 (22.7) | 338 (23.7) | 55.0 | 0.4025 |  |
| Preterm/low weight | 84 (11.1) | 65 (9.7) | 149 (10.4) | 56.4 | 0.3892 |  |
| Exclusive breastfeeding ≥ 3 months | 385 (50.8) | 347 (51.7) | 732 (51.2) | 52.6 | 0.7278 |  |
| Motor delay | 41 (5.4) | 32 (4.8) | 73 (5.1) | 56.2 | 0.5834 |  |
| Language delay | 165 (21.8) | 150 (22.4) | 315 (22.0) | 52.4 | 0.7894 |  |
| ADHD type |  |  |  |  |  |  |
| Inattentive | 213 (28.1) | 180 (26.8) | 393 (27.5) | 54.2 | 0.5902 |  |
| Combined | 484 (63.9) | 427 (63.6) | 911 (63.8) | 53.1 | 0.9325 |  |
| Hyperactive/impulsive | 61 (8.0) | 64 (9.5) | 125 (8.7) | 48.8 | 0.3196 |  |
| CGI-S score 5-7 at diagnosis | 249 (32.8) | 199 (29.7) | 448 (31.4) | 55.6 | 0.1942 |  |
| Comorbidities |  |  |  |  |  |  |
| Psychiatric comorbidities | -69.3 | 471 (70.2) | 996 (69.7) | 52.7 | 0.7018 |  |
| Chronic diseases | 46 (6.1) | 34 (5.1) | 80 (5.6) | 57.5 | 0.4111 |  |
| ***Therapy*** |  |  |  |  |  |  |
| At least one therapy | 649 (85.6) | 511 (76.2) | 1160 (81.2) | 55.9 | <0.0001 | * |
| Combined | 150 (19.8) | 79 (11.8) | 229 (16.0) | 65.5 | <0.0001 | * |
| Methylphenidate alone | 98 (12.9) | 82 (12.2) | 180 (12.6) | 54.4 |  |  |
| Psychological alone­ | 401 (52.9) | 350 (52.2) | 751 (52.6) | 53.4 |  |  |
| None | 109 (14.4) | 160 (23.8) | 269 (18.8) | 40.5 |  |  |

• Test χ^2^ used for categorical variables and Wilcoxon for continuous variables.

*Statistically significant

**Supplementary Table 2.** Propensity score calculation: results of the stepwise selection

| **Characteristics** | **Combined** | **Methylphenidate**  **alone** | **Psychological**  **alone** |
| --- | --- | --- | --- |
| ***Center*** |  |  |  |
| In centers with clinical staff professionals  over the median | 1.09 (1.01 - 1.19) | 1.02 (0.93 - 1.11) | 1.23 (1.16 - 1.30) |
| In centers with hours/year/patient of work  over the median | 0.66 (0.44 - 1.00) | 1.20 (0.77 - 1.87) | 1.01 (0.73 - 1.39) |
| Time from the request to diagnosis  over the median (days) | 1.00 (1.00 - 1.00) | 0.99 (0.99 - 1.00) | 1.00 (1.00 - 1.00) |
| ***Patients*** |  |  |  |
| Age at diagnosis | 1.14 (1.05 – 1.23) | 1.23 (1.13 - 1.34) | 0.88 (0.83 - 0.94) |
| Incomplete diagnostic evaluation: Yes vs No | 0.77 (0.39 - 1.51) | 0.77 (0.38 - 1.53) | 0.42 (0.24 - 0.75) |
| Breastfeeding exclusive ≥ 3 months | 1.19 (0.79 - 1.78) | 1.18 (0.77 - 1.81) | 1.56 (1.15 - 2.11) |
| ADHD type: H/I vs C | 0.30 (0.19 - 0.47) | 0.25 (0.15 - 0.41) | 0.84 (0.62 - 1.14) |
| CGI-S score at diagnosis: 5-7 vs 3-4 | 10.28 (6.52 - 16.21) | 6.72 (4.19 - 10.78) | 1.21 (0.82 - 1.80) |

**Supplementary Table 3.** Homogeneity test of treatment groups’ weights obtained by propensity score model

| **Therapy** | **Patients (N)** | **%** | **Included** | **Sum of the weights** | **% weights** | **p*** |
| --- | --- | --- | --- | --- | --- | --- |
| Combined | 229 | 16.0 | 229 | 1476.3 | 25.5 | 0.5042 |
| Methylphenidate alone | 180 | 12.6 | 180 | 1422.5 | 24.6 |  |
| Psychological alone | 751 | 52.6 | 751 | 1415.3 | 24.4 |  |
| None | 269 | 18.8 | 269 | 1477.5 | 25.5 |  |
| **Total** | **1429** | **100** | **1429** | **5791.7** | **100** |  |

* Test χ^2^ used for homogeneity of proportions.

**Supplementary Table 4.** Characteristics of the patients with improvements

| **Characteristics** | **Model M1** | **Model M2** | **Model M3** |
| --- | --- | --- | --- |
| ***Centers*** |  |  |  |
| Clinical staff professionals  over the median: No vs Yes |  | n.s. | n.s. |
| Hours/year/patient of work  over the median: No vs Yes |  | n.s. | n.s. |
| Time from the request to diagnosis | n.s. | n.s. | n.s. |
| ***Patients*** |  |  |  |
| Age at diagnosis | n.s. | n.s. | n.s. |
| Sex: Male vs Female | n.s. | n.s. | 0.71 (0.52 - 0.99) |
| Only child: Yes vs No | n.s. | n.s. | n.s. |
| Born abroad: Yes vs No | n.s. | n.s. | n.s. |
| Adopted: Yes vs No | n.s. | n.s. | n.s. |
| School failures: Yes vs No | n.s. | n.s. | n.s. |
| Employed parents: Yes vs No | n.s. | n.s. | n.s. |
| Family history of ADHD: Yes vs No | n.s. | n.s. | n.s. |
| Dystocic delivery: Yes vs No | n.s. | n.s. | n.s. |
| Preterm/low weight: Yes vs No | n.s. | n.s. | n.s. |
| Exclusive breastfeeding ≥ 3 months: Yes vs No | n.s. | n.s. | n.s. |
| Motor delay: Yes vs No | n.s. | n.s. | n.s. |
| Language delay: Yes vs No | n.s. | n.s. | n.s. |
| ADHD type: H/I vs. C | n.s. | n.s. | n.s. |
| CGI-S score at diagnosis: 5-7 vs 3-4 | n.s. | n.s. | n.s. |
| Comorbidities |  |  |  |
| Psychiatric comorbidities: Yes vs No | n.s. | n.s. | n.s. |
| Chronic diseases: Yes vs No | n.s. | n.s. | n.s. |
| ***Therapy*** |  |  |  |
| **Combined therapy vs None** |  |  | 3.07 (2.00 - 4.71) |
| Methylphenidate alone therapy vs None |  |  | 1.89 (1.22 - 2.92) |
| Psychological alone therapy vs None |  |  | 1.80 (1.32 - 2.46) |

Generalized linear mixed models with center as the random effect.

**Supplementary Table 5.** Log-odds ratio between observed and expected improvement by center (unadjusted)

| **Center** | **Observed (N)** | **Expected (N)** | **Total (N)** | **Log-odds (IC 95%)** |  |
| --- | --- | --- | --- | --- | --- |
| F | 35 | 25 | 48 | 0.91 (0.34 - 1.69) | * |
| K | 29 | 21 | 40 | 0.87 (0.25 - 1.74) | * |
| P | 36 | 29 | 54 | 0.54 (0.02 - 1.19) | * |
| O | 31 | 25 | 47 | 0.53 (-0.03–1.23) |  |
| E | 113 | 95 | 179 | 0.41 (0.12 - 0.73) | * |
| R | 15 | 13 | 24 | 0.34 (-0.44 - 1.34) |  |
| L | 73 | 73 | 137 | 0.00 (-0.34 - 0.34) |  |
| I | 33 | 33 | 63 | 0.00 (-0.50 - 0.51) |  |
| M | 132 | 134 | 252 | -0.03 (-0.28 - 0.22) |  |
| Q | 81 | 83 | 156 | -0.05 (-0.37 - 0.27) |  |
| D | 20 | 21 | 39 | -0.10 (-0.75 - 0.55) |  |
| H | 18 | 19 | 35 | -0.11 (-0.80 - 0.58) |  |
| J | 65 | 84 | 159 | -0.48 (-0.8- -0.17) | * |
| B | 17 | 22 | 41 | -0.49 (-1.17-0.12) |  |
| C | 34 | 45 | 85 | -0.52 (-0.98- -0.10) | * |
| A | 26 | 37 | 70 | -0.64 (-1.17- -0.18) | * |
| **Total** | **758** | **758** | **1429** |  |  |

*Statistically significant

**Supplementary Table 6.** Log-odds ratios between observed and expected improvement by center (adjusted by Model 3)

| **Center** | **Observed (N)** | **Expected (N)** | **Total (N)** | **Log-odds (IC 95%)** |  |
| --- | --- | --- | --- | --- | --- |
| O | 31 | 21 | 47 | 0.85 (0.29 - 1.55) | * |
| K | 29 | 24 | 40 | 0.59 (-0.03 - 1.46) |  |
| P | 36 | 29 | 54 | 0.56 (0.03 - 1.21) | * |
| F | 35 | 35 | 48 | 0.47 (-0.10 - 1.26) |  |
| E | 113 | 95 | 179 | 0.43 (0.13 - 0.74) | * |
| R | 15 | 14 | 24 | 0.18 (-0.60 - 1.18) |  |
| Q | 81 | 78 | 156 | 0.07 (-0.24 - 0.39) |  |
| M | 132 | 134 | 252 | -0.03 (-0.27 - 0.22) |  |
| H | 18 | 19 | 35 | -0.12 (-0.80 - 0.58) |  |
| I | 33 | 35 | 63 | -0.16 (-0.66 - 0.35) |  |
| B | 17 | 20 | 41 | -0.28 (-0.96 - 0.33) |  |
| D | 20 | 23 | 39 | -0.30 (-0.94 - 0.36) |  |
| L | 73 | 84 | 137 | -0.31 (-0.65 - 0.03) |  |
| J | 65 | 78 | 159 | -0.33 (-0.65 - -0.02) | * |
| C | 34 | 47 | 85 | -0.62 (-1.08 - -0.19) | * |
| A | 26 | 37 | 70 | -0.64 (-1.17 - -0.18) | * |
| **Total** | **758** | **770** | **1429** |  |  |

*Statistically significant

**Supplementary Figure 1.** Clinical improvement (CGI-I ≤ 3) according to the treatment received (Std. Res.). *Statistically significant.


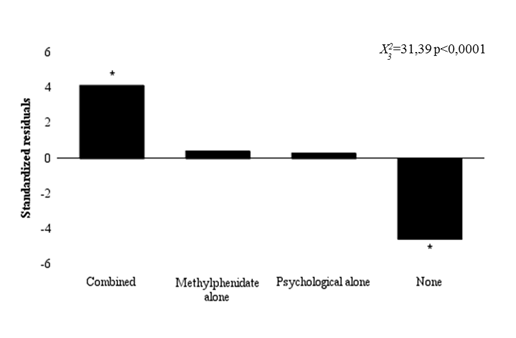

Supplement: Supplementary file 1 — Supplementary Information. [file 41598_2023_45714_MOESM1_ESM.docx]
